# Bowel Management and Standard Urotherapy in Pediatric Bladder and Bowel Dysfunction: A Randomized Clinical Trial

**DOI:** 10.1001/jamanetworkopen.2026.8836

**Published:** 2026-04-27

**Authors:** Sofie Axelgaard, Konstantinos Kamperis, Søren Hagstrøm, Lien Dossche, Ann-Kristine Mandøe Svendsen, Lise Fischer Mikkelsen, Søren Isidor, Bolette Brodersen, Luise Borch

**Affiliations:** 1 Department of Paediatrics and Adolescent Medicine, Gødstrup Hospital, Herning, Denmark; 2 Department of Clinical Medicine, Aarhus University, Aarhus, Denmark; 3 NIDO, Centre for Research and Education, Gødstrup Hospital, Herning, Denmark; 4 Department of Paediatrics and Adolescent Medicine, Aarhus University Hospital, Aarhus, Denmark; 5 Department of Paediatrics and Adolescent Medicine, Aalborg University Hospital, Aalborg, Denmark; 6 Department of Paediatric Nephrology, Ghent University Hospital, Ghent, Belgium; 7 Steno Diabetes Center Aarhus, Aarhus University Hospital, Aarhus, Denmark

## Abstract

**Importance:**

Bladder and bowel dysfunction is a common pediatric condition characterized by functional constipation and lower urinary tract symptoms, including daytime urinary incontinence. It increases the risk of urinary tract infections and kidney damage and imposes substantial psychosocial burden. High-quality evidence regarding optimal sequencing of bowel and bladder interventions is limited.

**Objective:**

To examine whether adding standard urotherapy to bowel management further reduces daytime urinary incontinence in children with bladder and bowel dysfunction.

**Design, Setting, and Participants:**

This multicenter, open-label, parallel-group randomized clinical trial was conducted from September 1, 2022, to July 31, 2025, with 12-week follow-up at 5 outpatient pediatric clinics in Denmark. Children aged 5 to 14 years with treatment-naive bladder and bowel dysfunction were included in the modified intention-to-treat analysis.

**Interventions:**

Twelve weeks of bowel management alone (monotherapy) or combined with standard urotherapy (combination therapy). Bowel management included disimpaction and maintenance therapy, toileting after meals, and caregiver education. Urotherapy included education, timed voiding, optimal toilet posture, and fluid intake guidance.

**Main Outcomes and Measures:**

The primary outcome was wet days per week, assessed by the Dry Pie bladder diary and reported as (1) risk of a wet day, (2) expected number of wet days per week, and (3) response rates: no (<50%), partial (50%-99%), or complete (100%) response.

**Results:**

Ninety-four children (median [IQR] age, 6.9 [6.1-8.2] years; 57 [60.6%] male) were randomized; 83 completed follow-up. At baseline, the daily risk of a wet day was 0.75 (95% CI, 0.70-0.80). After 12 weeks, risks were 0.54 (95% CI, 0.44-0.65) for monotherapy and 0.55 (95% CI, 0.45-0.66) for combination therapy. Expected wet days decreased from 5.3 (95% CI, 4.9-5.6) to 3.8 (95% CI, 3.0-4.6) and 3.9 (95% CI, 3.1-4.6). The number of participants achieving a 50% or greater reduction in the number of wet days per week was 16 (38.1%; 95% CI, 23.6%-54.4%) in the monotherapy group and 12 (29.3%; 95% CI, 16.1%-45.5%) in the combination therapy group. Complete response was achieved by 6 (14.3%; 95% CI, 5.4%-28.5%) in the monotherapy group and 4 (9.8%; 95% CI, 2.7%-23.1%) in the combination therapy group. Constipation resolution was associated with larger reductions (expected 3.44 vs 4.15 per week; adjusted mean difference, −0.89; 95% CI, −1.94 to −0.05).

**Conclusions and Relevance:**

In this randomized clinical trial of children with bladder and bowel dysfunction, bowel management reduced daytime urinary incontinence; concurrent urotherapy provided no additional benefit. This finding highlights that bowel management alone was an effective first-line intervention and may be a prerequisite for successful standard urotherapy.

**Trial Registration:**

ClinicalTrials.gov NCT05318365

## Key Points

**Question:**

Does adding standard urotherapy to bowel management improve daytime urinary incontinence in children with bladder and bowel dysfunction?

**Findings:**

In this randomized clinical trial of 94 children, 12 weeks of bowel management reduced the risk of a wet day from 0.75 to 0.54 and to 0.55 when combined with urotherapy. Children achieving constipation resolution had greater reductions.

**Meaning:**

These findings suggest that bowel management alone is an effective first-line intervention for daytime urinary incontinence in children with bladder and bowel dysfunction, and adding urotherapy at treatment initiation does not provide additional benefit.

## Teaser

This randomized clinical trial evaluates whether daytime urinary incontinence is improved with the combination of standard urotherapy and bowel management vs bowel management alone in children with bladder and bowel dysfunction.

## Introduction

Bladder and bowel dysfunction (BBD) is defined by the coexistence of functional constipation and lower urinary tract symptoms, including urgency, altered voiding frequency, daytime urinary incontinence (DUI), and nocturnal enuresis in children 5 years or older.^1,2^ BBD is common yet frequently underdiagnosed, affecting up to 20% of 7-year-olds^3,4^ and accounting for approximately 40% of pediatric urology referrals.^5^ BBD increases the risk of upper urinary tract infections,^6,7,8,9^ kidney scarring,^10^ chronic kidney disease, and hypertension^11,12^ and adversely affects self-esteem and quality of life.^13,14^

Anatomical and neurophysiologic interactions between the bladder and bowel contribute to symptom overlap.^15,16^ Consequently, current treatment strategies adopt a stepwise approach, prioritizing bowel management before lower urinary tract symptom–specific interventions.^17,18^ Bowel management includes education, disimpaction, maintenance therapy, and scheduled toileting after meals.^1^ Lower urinary tract symptom–specific therapy includes standard urotherapy (SU), emphasizing timed voiding every 2 to 3 hours, optimal posture, discouragement of holding behaviors, and adequate fluid intake.^19,20,21^

When initial interventions are insufficient, the International Children’s Continence Society (ICCS) recommends targeted urotherapy, such as pelvic floor retraining, biofeedback, neuromodulation, and intermittent catheterization, or pharmacologic treatment, typically beginning with anticholinergics.^1,2^ The initial stepwise treatment approach is supported by limited evidence, with only a few studies suggesting that bowel management alone may reduce DUI in affected children.^22,23^ Current recommendations regarding sequencing of treatment modalities, including those endorsed by the European Association of Urology and the European Society of Paediatric Urology, supported by the ICCS are based on low-level evidence.^17,18^ Thus, high-quality prospective data to guide the optimal order of therapeutic components for children with BBD are highly warranted. This randomized clinical trial (RCT) aimed to evaluate whether combining SU with bowel management improves DUI compared with bowel management alone in children with BBD.

## Methods

### Study Design and Participants

This multicenter, open-label, parallel-group RCT enrolled children aged 5 to 14 years with treatment-naive BBD from 5 hospital outpatient clinics across Denmark (Aarhus, Aalborg, Gødstrup, Viborg, and Kolding) via primary care referrals and social media from September 1, 2022, to July 31, 2025, with 12-week follow-up. Eligibility required functional constipation (≥2 Rome IV criteria) and DUI at least twice weekly for 1 month or longer. Exclusion criteria included known anatomical or neurologic urinary or gastrointestinal abnormalities, prior or current urotherapy, or pharmacologic DUI treatment. Children receiving laxative monotherapy were eligible if constipation remained uncontrolled. Full criteria are in eTable 1 in Supplement 1. The trial was planned according to SPIRIT 2013 guidelines^22^ and is reported per the Consolidated Standards of Reporting Trials (CONSORT) reporting guidelines.^23^ The full trial protocol and statistical analysis plan are available in Supplement 2. The study was approved by the Regional Committee on Health Research Ethics of the Central Denmark Region and registered at ClinicalTrials.gov. Written informed consent was obtained from the parents or legal guardians before enrollment.

### Randomization and Masking

Participants were block-randomized 1:1 within each study site using variable block sizes^2,3,4,5,6,7,8^ via the electronic REDCap (Research Electronic Data Capture) portal, which automatically concealed the allocation sequence.^24,25^ Study personnel who enrolled participants did not have access to future allocations. Due to the behavioral nature of SU, participants and caregivers were not blinded. Outcome assessors and data analysts were aware of group assignments.

### Intervention

Participants received 12 weeks of individually tailored bowel management according to the European Society for Paediatric Gastroenterology, Hepatology, and Nutrition guidelines,^26^ alone or combined with SU. Bowel management included disimpaction with polyethylene glycol (1.0-1.5 g/kg daily for ≥3 days) or a single dose of sodium picosulfate if polyethylene glycol was not feasible. Maintenance therapy with polyethylene glycol (0.5-0.8 g/kg daily) or magnesium hydroxide (500-1000 mg/d) was adjusted for response and tolerance. Stimulant laxatives (sodium picosulfate) or transanal irrigation were added as appropriate. Scheduled toileting after meals was performed 1 to 3 times daily.

SU included education of the child and caregivers, instruction on optimal toilet posture, adequate fluid intake (1200-1500 mL/d), and timed voiding supported by a timer watch, usually every 2 hours. Adherence to bowel management was monitored in all participants, with laxative doses adjusted as needed through telephone consultations at weeks 2 and 4. In the SU arm, adherence to urotherapy was reinforced during these contacts. Outcomes were evaluated at week 12 and compared with baseline. Adverse events were recorded as part of routine clinical practice; no new interventions were introduced.

### Outcomes

#### Primary End Point

The primary outcome was the number of wet days per week, assessed by the Dry Pie bladder diary (eFigure in Supplement 1).^27^ Participants, with age-appropriate assistance from caregivers, completed the diary for the week prior to each study visit. Each day was color-coded to reflect the most severe episode of incontinence. Treatment response was assessed by the estimated daily risk of a wet day, the expected number of wet days per week, and response rates according to ICCS guidelines,^2^ defined as follows: (1) no response (<50% reduction in the number of wet days), (2) partial response (50%-99% reduction in the number of wet days), and (3) complete response (100% reduction in the number of wet days [complete dryness during the diary week]).

#### Secondary End Points

Secondary outcomes included incontinence severity, bowel outcomes, and bladder function. Incontinence severity was quantified using a score derived from the Dry Pie diary.^28^ Each day was scored as follows: 0, dry day; 1, incontinence limited to wet underclothing; 2, wet clothes; and 3, wet socks. Bowel outcomes included stool consistency (Bristol Stool Scale), rectal diameter assessed by abdominal ultrasonography,^29^ and the number of fulfilled Rome IV criteria. Fecal incontinence was reported both within the Rome IV framework and as a separate outcome due to its clinical significance. Bladder function was assessed using frequency volume medical record–derived measures: daily voiding frequency and maximum voided volume relative to expected bladder capacity, considered a proxy for functional bladder volume.

### Post Hoc Analyses

 To evaluate the impact of bowel management on DUI, we pooled all participants irrespective of randomization and categorized according to bowel management outcome at follow-up: successful (<2 Rome IV criteria) and nonsuccessful (≥2 Rome IV criteria). The primary outcome, wet days per week, was analyzed both unadjusted and adjusted for baseline wet days. To evaluate the effect of adherence to urotherapy, we categorized participants in the SU arm into 3 adherence groups: full (n = 16), partial (n = 17), or none (n = 8). Adherence was assessed at visit 2 and during telephone follow-ups. Those who performed no urotherapy the week prior to visit 2 were considered nonadherent. Participants missing more than 1 alarm per day were partially adherent; those adhering during telephone follow-ups and missing 1 or fewer alarms per day at visit 2 were fully adherent.

### Statistical Analysis 

Sample size calculations were based on previously reported treatment response rates.^21,30^ A total of 82 participants (41 per group) were required to detect a clinically meaningful difference in complete dryness rates with 80% power at a 2-sided α = .05. The final analyzed cohort included 83 participants; details are provided in the eMethods and eAppendix in Supplement 1. Baseline characteristics were summarized descriptively. Continuous variables were assessed for normality using Q-Q plots and reported as mean (SD) or median (IQR) as appropriate. Categorical variables were presented as numbers (percentages).

Primary and secondary outcomes were analyzed in the modified intention-to-treat (mITT) population (all randomized participants with 12-week follow-up); participants with missing data were included in baseline summaries but excluded from analyses, without imputation. Daily risk of DUI was estimated using binomial regression with robust variance clustered by participant; from this, the expected number of wet days per week was derived. Effect estimates are reported as risk ratios and risk differences with 95% CIs. Response rates were compared using χ^2^ tests, with relative risks (RRs) and 95% CIs. Incontinence severity scores were analyzed using analysis of covariance, with model assumptions evaluated by residual diagnostics; results are presented as adjusted mean differences with 95% CIs.

No formal analyses of adverse events were performed; events were monitored as part of routine clinical care, and no unexpected or clinically significant events occurred. All analyses were performed in R, version 4.5.1 (R Foundation for Statistical Computing), with 2-sided *P* < .05 considered statistically significant.

## Results

### Participants and Clinical Characteristics

**Figure 1.**
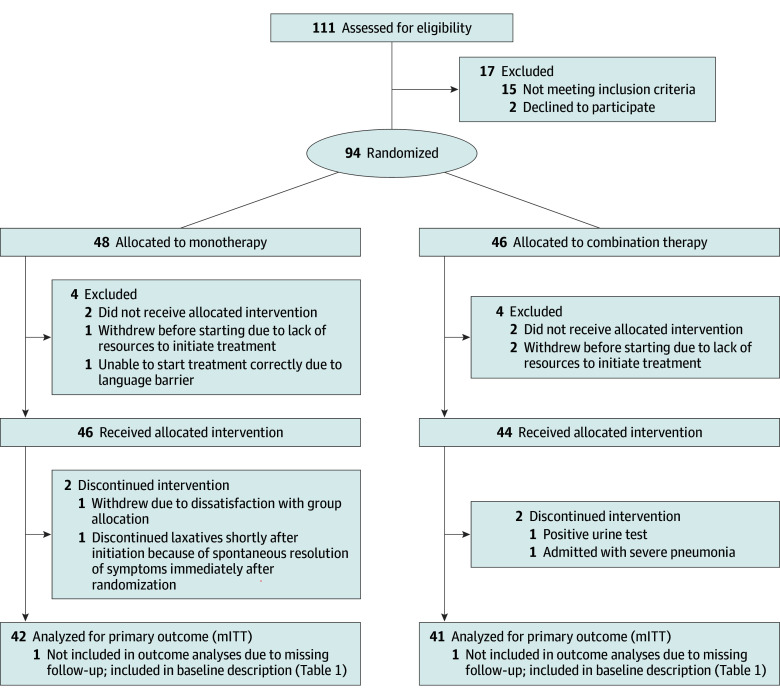
CONSORT Flow Diagram Standard CONSORT flow diagram showing the number of participants assessed for eligibility, randomized, allocated to each treatment group, unavailable for follow-up, and included in the primary analysis. mITT indicates modified intention to treat. CONSORT indicates Consolidated Standards of Reporting Trials.

Ninety-four children (median [IQR] age, 6.9 [6.1-8.2] years; 57 [60.6%] male and 37 [39.4%] women) were randomized to bowel management alone (monotherapy) or bowel management combined with SU (combination therapy) (Figure 1). Eleven discontinued before end point assessment, leaving 83 for the mITT analysis. Reasons in the monotherapy group (n = 6) included withdrawal before starting treatment (n = 2), dissatisfaction with allocation (n = 1), spontaneous symptom resolution (n = 1), and missing follow-up data (n = 2). In the combination therapy group (n = 5), reasons included withdrawal before starting treatment (n = 2), clinical issues (positive urine culture result or hospital admission, n = 2), and missing follow-up data (n = 1).

All 94 participants were included in the intention-to-treat (ITT) population for baseline characteristics, which were generally balanced (Table 1). Baseline characteristics for all populations, including ITT, mITT total, and mITT by treatment allocation, are given in eTable 2 in Supplement 1. No other concurrent interventions relevant to bowel or bladder function were administered during the study period. No unexpected or clinically significant adverse events occurred.

**Table 1.** Patient Characteristics by Treatment Group (Intention-to-Treat Population)

Characteristic	No. (%) of patients^a^	
	Monotherapy (n = 48)	Combination therapy (n = 46)
Sex		
Male	30 (62.5)	27 (58.7)
Female	18 (37.5)	19 (41.3)
Age, median (IQR), y	7.0 (6.2-8.1)	6.8 (6.0-8.6)
BMI, median (IQR)	16.1 (15.1-18.0)	16.2 (14.8-17.2)
Previous UTI	5 (10.4)	2 (4.3)
Medicine at treatment onset	8 (16.7)	12 (26.1)
Polyethylene glycol	6 (12.5)	11 (23.9)
Sodium picosulfate	1 (2.1)	0
PPI	0	1 (2.4)
Fluticasone furoate	1 (2.1)	0
Psychiatric disorder or under evaluation	3 (6.3)	5 (10.9)
ADHD	2 (4.2)	4 (8.7)
Anxiety	1 (2.1)	1 (2.2)
Nocturnal enuresis	35 (72.9)	24 (55.8)
Secondary DUI^b^	13 (27.7)	7 (17.1)

Abbreviations: ADHD, attention-deficit/hyperactivity disorder; BMI, body mass index (calculated as weight in kilograms divided by the square of height in meters); DUI, daytime urinary incontinence; PPI, proton pump inhibitor; UTI, urinary tract infection.

^a^ Unless otherwise indicated.

^b^ Secondary DUI refers to children who have previously experienced at least 6 months of continence.

### Bowel Management

At baseline, 83 of 94 participants (88.3%) received a bowel cleanout (polyethylene glycol or sodium picosulfate), 9 (9.6%) maintenance stool softeners only (polyethylene glycol or magnesium hydroxide), and 2 (2.1%) stimulant laxatives. At follow-up, 78 of 83 (94.0%) continued maintenance stool softeners, 5 (6.0%) used stimulant laxatives, and 9 (10.8%) received low-volume irrigation; some participants received combination treatments, explaining percentages exceeding 100% (eTable 3 in Supplement 1). Interventions were delivered according to protocol, with high adherence to bowel management and variable adherence to SU as reported.

### Effect on DUI

**Figure 2.**
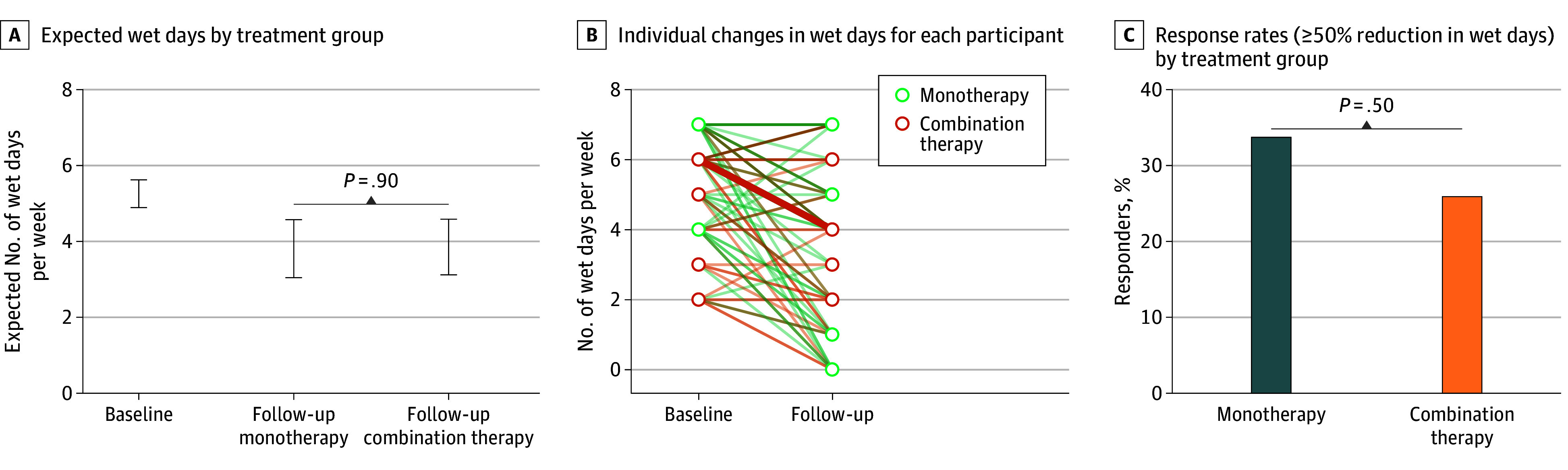
Expected Wet Days and Response by Treatment Group (Modified Intention-to-Treat [mITT] Population) A, Error bars indicate 95% CIs. B, A spaghetti plot of individual participant trajectories. Each line represents 1 or more participants, and the line thickness indicates the number of participants with identical or overlapping values at that point.

Model-based estimates showed that the daily risk of a wet day decreased from 0.75 (95% CI, 0.70-0.80) at baseline to 0.54 (95% CI, 0.44-0.65) in the monotherapy group and 0.55 (95% CI, 0.45-0.66) in the combination therapy group. The expected number of wet days per week decreased from 5.3 (95% CI, 4.9-5.6) to 3.8 (95% CI, 3.0-4.6) and 3.9 (95% CI, 3.1-4.6), respectively, with no between-group difference (*P* = .92) (Table 2 and Figure 2). The number of participants achieving a 50% or greater reduction in the number of wet days per week was 16 (38.1%; 95% CI, 23.6%-54.4%) in the monotherapy group and 12 (29.3%; 95% CI, 16.1%-45.5%) in the combination therapy group, again with no between-group difference (RR, 0.88; 95% CI, 0.64-1.19; *P* = .54) (Table 2 and Figure 2). Complete response was achieved by 6 (14.3%; 95% CI, 5.4%-28.5%) in the monotherapy group and 4 (9.8%; 95% CI, 2.7%-23.1%) in the combination therapy group. Mean (SD) incontinence severity scores decreased from 8.2 (3.3) at baseline to 5.8 (4.4) at follow-up in the monotherapy group and from 7.6 (4.1) to 5.5 (3.9) in the combination therapy group. The estimated mean difference between groups at follow-up (combination therapy vs monotherapy) was –0.1 (95% CI, –0.7 to 0.5; *P* = .71).

**Table 2.** Estimated Risk of a Wet Day and Expected Number of Wet Days Per Week at Baseline and Follow-Up According to Treatment Group (Modified Intention-to-Treat Population)

Outcome	Baseline overall^a^ (N = 83)	Follow-up monotherapy^b^ (n = 42)	Follow-up combination therapy^b^ (n = 41)	*P* value (monotherapy vs combination therapy)^c^
Daily risk of a wet day (95% CI)	0.75 (0.70-0.80)	0.54 (0.44-0.65)	0.55 (0.45-0.66)	.92
Expected No. of wet days (95% CI)	5.3 (4.9-5.6)	3.8 (3.0-4.6)	3.9 (3.1-4.6)	.92

^a^ Baseline values represent model-based estimates for the total study population before randomization.

^b^ Follow-up values are predicted probabilities or expected numbers from binomial regression models.

^c^ *P* values indicate between-group differences at follow-up.

### Other Bladder and Bowel Outcomes

Secondary outcomes are listed in eTable 4 in Supplement 1. Fecal incontinence decreased, rectal diameter tended to reduce, whereas Bristol Stool Scale and voiding frequency remained stable. Fluid intake increased in SU participants, as expected. The maximum voided volume relative to expected bladder capacity tended to be lower at baseline in the monotherapy group, but differences were not significant. Urge prevalence showed minor changes.

### Impact of Successful Bowel Management

At follow-up, children with constipation resolution had 3.44 wet days per week (95% CI, 2.72-4.16) compared with 4.15 wet days (95% CI, 3.39-4.91) in those without resolution (Table 3). After adjusting for baseline wet days, the adjusted mean difference was –0.89 wet days (95% CI, –1.94 to –0.05; *P* = .04), with an adjusted daily RR of 0.78 (95% CI, 0.61-0.99; *P* = .04). Unadjusted analyses showed no statistically significant effect (mean difference, –0.71; 95% CI, –1.76 to 0.34; RR, 0.83; 95% CI, 0.63-1.19).

**Table 3.** Estimated Risk of a Wet Day and Expected Number of Wet Days Per Week at Baseline and Follow-Up, According to Successful vs Unsuccessful Bowel Management (Modified Intention-to-Treat Population)^a^

Outcome	Unsuccessful bowel management (n = 41)	Successful bowel management (n = 42)	Effect estimate (95% CI)	*P* value
Daily risk of wet day	0.59 (0.48 to 0.70)	0.49 (0.39 to 0.59)	RR, 0.83 (−0.63 to 1.19)	.19
Wet days per week	4.15 (3.39 to 4.91)	3.44 (2.72 to 4.16)	MD, −0.71 (–1.76 to 0.34)	.19
Daily risk of wet day^b^	NA	NA	RR, 0.78 (0.61 to 0.99)	.04^c^
Wet days per week^b^	NA	NA	MD, –0.89 (–1.94 to –0.05)	.04^c^

Abbreviations: MD, mean difference; NA, not applicable; RR, risk ratio.

^a^ Successful is defined as less than 2 Rome IV criteria at follow-up; unsuccessful, 2 or more Rome IV criteria at follow-up.

^b^ Adjusted for baseline number of wet days.

^c^ Statistically significant (*P* < .05). Estimates are predicted marginal means from binomial regression models.

### Adherence to SU

At follow-up, the unadjusted daily risk of wet days was 0.52 (95% CI, 0.36-0.68) in the full adherence group, 0.66 (95% CI, 0.50-0.82) in the partial group, and 0.39 (95% CI, 0.17-0.61) in the no adherence group, with no group differing from monotherapy. Adjustment for baseline wet days did not change the findings.

## Discussion

In this RCT of children with BBD, bowel management significantly improved daytime urinary incontinence, whereas concurrent SU did not confer additional benefit. No unexpected or clinically significant adverse events occurred.

### Bowel Management and DUI

The association between successful bowel management and improvement of DUI was first described in 1997 by Loening-Baucke et al,^31^ who treated 234 children with chronic constipation for at least 12 months. Among the 29% with concomitant DUI, complete resolution occurred in 73% and in 89% of those with successful relief of constipation. Borch et al^30^ later confirmed this association, reporting a 50% or greater reduction in DUI in 68% and complete resolution in 27% of children with BBD.

Response rates in our trial were lower, likely reflecting the shorter follow-up (12 weeks vs ≥12 months in the study by Loening-Baucke et al^31^ and 4 months in the study by Borch et al^30^) and the inclusion of all children with constipation, whereas the study by Borch et al^30^ included only those successfully treated.

Several mechanisms may underlie the association between bowel dysfunction and lower urinary tract symptoms. Mechanical factors, such as rectal distension and fecal impaction, may exert pressure on the bladder or alter the vesicourethral angle, provoking detrusor overactivity, urgency, or incomplete emptying.^15,32^ Emerging evidence suggests a more complex interaction involving sensoneurologic pathways: rectal distension inhibits bladder contractions,^33^ and animal models demonstrate cross-talk between colonic and bladder innervation at the lumbosacral cord level.^34^

Central mechanisms are increasingly recognized. Children with BBD more frequently present with neuropsychiatric comorbidities, including attention-deficit/hyperactivity disorder and affective disorders,^35,36,37,38,39^ and neuroimaging studies^40,41^ implicate the frontal cortex and anterior cingulate gyrus in both continence control and executive function. These findings support the hypothesis that BBD may partly reflect altered central regulation rather than isolated bladder or bowel dysfunction.

Our study cannot distinguish mechanical from sensorineural or central mechanisms. However, the modest treatment effect after 12 weeks may reflect the contribution of sensory and central disturbances, which may require a longer time to normalize, and may be more pronounced in children with neuropsychiatric comorbidities.^33,34,39,42,43,44^

### SU and BBD

A substantial body of evidence supports the efficacy of SU in children with DUI. In a retrospective cohort of 240 children, 55% achieved continence with SU, increasing to 70% among those receiving timer-watch support.^21^ In a 12-week prospective trial using systematic timer-watch assistance, partial and full response rates were 60% and 30%, respectively.^41^ These estimates align with a 2018 meta-analysis,^20^ and several studies show that timer-watch assistance enhances SU effects.^45,46^

SU has not been systematically studied in children with BBD outside concomitant bowel management, making it difficult to disentangle the effects of each intervention. In our trial, the addition of SU to bowel management did not confer an additional benefit. Several explanations are possible. First, bladder and bowel symptoms may arise from overlapping neural mechanisms rather than additive processes^47^ so that normalization of bowel function alone can suffice to improve continence. Second, persistent constipation may attenuate responsiveness to urotherapy because rectal distension and altered sensory pathways can reduce the efficacy of bladder training.^32^ These findings underscore the importance of adequately treating constipation as a prerequisite for optimizing the effect of SU. Third, despite randomization, children in the combination therapy group tended to have lower functional bladder volumes, potentially limiting the scope for improvement, consistent with the inverse relationship between bladder overactivity and response to SU.^48^

### Adherence to SU

Despite timer-watch support and telephone calls to ensure adherence, adherence to SU was suboptimal: 80.5% continued SU at follow-up, with only 39.0% reporting full adherence. In comparison, Hagstrøm et al^49^ reported adherence rates of 67% in children with DUI undergoing timer-watch–assisted urotherapy, a modality associated with higher adherence compared with urotherapy without a timer watch (RR, 2.97; 95% CI, 1.46-6.06).^46^

The current study did not demonstrate any differences in treatment responses according to SU adherence. Although not statistically significant, less severe DUI in children who discontinued SU suggests motivation may be lower among those with milder symptoms. Reduced adherence to SU among children with BBD compared with those with isolated DUI may also reflect the added treatment burden of managing both bowel and bladder regimens, the higher prevalence of psychiatric comorbidities,^36^ and possibly limited perceived efficacy of SU when constipation remains inadequately controlled.

### Limitations

Limitations of this study include the relatively short 12-week follow-up and the lack of long-term outcomes. No untreated control group was included, because withholding bowel management for 12 weeks would have been unethical. Consequently, observed effects may partly reflect placebo, the natural history of DUI (which generally shows lower responses in comparable age groups^50^), or regression to the mean.^51^ Primary analyses were conducted in the mITT population, excluding participants without follow-up data; however, attrition was low and balanced between groups, and baseline characteristics of the ITT and mITT populations were similar, suggesting minimal impact on the results. Absence of an SU-only arm limits interpretation of whether lower efficacy reflects the inherent complexity of BBD or the added burden of dual interventions.

Future studies should evaluate SU both before and after effective bowel management to clarify the temporal sequence of bladder-bowel interactions. Incorporating functional imaging may further elucidate underlying mechanisms and inform more targeted interventions.

## Conclusions

This RCT found that bowel management significantly reduced DUI in children with BBD and that adding SU at treatment initiation provided no additional benefit. This finding highlights that bowel management alone was an effective first-line intervention and may be a prerequisite for successful SU.

## Notes

Supplement 1. **eAppendix.** Sample Size Calculation **eTable 1.** Inclusion and Exclusion Criteria **eTable 2.** Baseline Characteristics Across ITT and mITT Populations, Stratified by Treatment Group **eTable 3.** Laxatives Prescribed During the Study Period **eTable 4.** Secondary Outcomes **eFigure.** Dry Pie Bladder Diary

Supplement 2.
Trial Protocol and Statistical Analysis Plan

Supplement 3.
Data Sharing Statement
