# Supplementary material for: Bowel Management and Standard Urotherapy in Pediatric Bladder and Bowel Dysfunction: A Randomized Clinical Trial
Source: JAMA Netw Open. 2026 Apr 27;9(4):e268836. doi: 10.1001/jamanetworkopen.2026.8836 (PMC13122393; doi:10.1001/jamanetworkopen.2026.8836)
Supplement: Supplement 1. — eAppendix. Sample Size Calculation eTable 1. Inclusion and Exclusion Criteria eTable 2. Baseline Characteristics Across ITT and mITT Populations, Stratified by Treatment Group eTable 3. Laxatives Prescribed During the Study Period eTable 4. Secondary Outcomes eFigure. Dry Pie Bladder Diary [file jamanetwopen-e268836-s001.pdf]

Supplemental Online Content

Axelgaard S, Kamperis K, Hagstrøm S, et al. Bowel management and standard urotherapy in pediatric bladder and bowel dysfunction: a randomized clinical trial. *JAMA Netw Open*. 2026;9(4):e268836. doi:10.1001/jamanetworkopen.2026.8836

eAppendix. Sample Size Calculation

eTable 1. Inclusion and Exclusion Criteria

eTable 2. Baseline Characteristics Across ITT and mITT Populations, Stratified by Treatment

Group

eTable 3. Laxatives Prescribed During the Study Period

eTable 4. Secondary Outcomes

eFigure. Dry Pie Bladder Diary

This supplemental material has been provided by the authors to give readers additional information about their work.

© 2026 Axelgaard S et al. *JAMA Network Open.*

eAppendix. Sample size calculation

Sample size was calculated based on previously reported treatment response rates. Borch et al. found that 68% of children with bladder and bowel dysfunction (BBD) achieved ≥50% reduction in wet days after bowel dysfunction management (1), with 27% achieving complete continence. Hagstrøm et al. reported that 55% of children became dry with standard urotherapy, including both children with isolated daytime urinary incontinence and BBD (2). Assuming 27% complete dryness in the monotherapy group (bowel management alone) and 55% in the combination therapy group (bowel management plus standard urotherapy), 82 participants (41 per group) were required to detect this difference with 80% power at a two-sided significance level of 0.05 using a chi-square test. To account for potential dropouts, recruitment was set at 100 participants. The final analyzed cohort of 83 participants was sufficient to detect clinically meaningful differences.

© 2026 Axelgaard S et al. *JAMA Network Open.*

eTable 1. Inclusion and Exclusion Criteria

**Inclusion criteria**	**Exclusion criteria**
Age 5 to 14 years	Known neurological or anatomical abnormalities of the kidneys, urinary tract, or gastrointestinal system
Diagnosis of daytime urinary incontinence (≥2 weekly episodes)	Previous surgical intervention involving the bowel or urinary tract (except circumcision)
Diagnosis of constipation according to Rome IV criteria (≥2 criteria)	Neurological disease or previous cerebral surgery
Normal objective clinical examination	Current urinary tract infection confirmed by positive urine culture
Parents or legal guardians able to understand written and oral study information	Ongoing or previous urotherapy
Informed consent obtained from both parents or legal guardians	Ongoing or previous treatment with anticholinergics and/or β3-receptor agonists
	Ongoing laxative treatment at an adequate dose (≥1 g/kg/day) unless constipation remains inadequately controlled, defined as still fulfilling at least two Rome IV criteria
	Inflammatory bowel disease
	Other known gastrointestinal, renal, or urinary tract diseases

© 2026 Axelgaard S et al. *JAMA Network Open.*

eTable 2. Baseline characteristics across ITT and mITT populations, stratified by treatment group

	ITT	mITT	mITT mono	mITT comb
N	94	83	42	41
Male sex, n (%)	57 (60.6)	52 (62.7)	26 (61.9)	26 (63.4)
Age (years), median (IQR)	6.9 (6.1-8.2)	6.9 (6.1-8.2)	7 (6.2-8.1)	6.8 (6-8.6)
BMI (kg/m²), median (IQR)	16.1 (15.1-17.5)	16.1 (15.1-17.2)	16.1 (15.1-17.9)	16.1 (15.2-17.1)
Previous UTI, n (%)	7 (7.4)	5 (6.0)	4 (9.5)	1 (2.4)
Medicine at treatment onset, n (%)	20 (21.3)	18 (21.7)	6 (14.3)	12 (29.3)
Psychiatric comorbidity, n (%)	8 (8.5)	8 (9.6)	3 (7.1)	5 (12.2)
Nocturnal enuresis, n (%)	59 (64.8)	54 (65.1)	30 (71.4)	24 (58.5)
Secondary DUI, n (%)	20 (22.7)	18 (22.5)	12 (29.3)	6 (15.4)

Abbreviations: UTI, urinary tract infection; DUI, daytime urinary incontinence.

© 2026 Axelgaard S et al. *JAMA Network Open.*

eTable 3. Laxatives subscribed during the study period

Type of laxatives	Baseline, n/N (%)	Follow-up, n/N (%)
Disimpaction (PEG 3350 or sodium picosulfate)	83/94 (88)	0/83 (0)
Stool softeners maintenance dose (PEG 3350 or magnesium hydroxide)	9/94 (10)	78/83 (94)
Stimulant laxatives (Sodium picosulfate)	2/94 (2)	5/83 (6)
Low-volume irrigation (Glyoktyl)	0/94 (0)	9/83 (11)

Note. Data are presented as n/N (%). PEG 3350 = polyethylene glycol 3350. Follow-up numbers reflect participants remaining in the study. Some participants received more than one type of laxative concurrently, so percentages may sum to >100% at follow-up.

© 2026 Axelgaard S et al. *JAMA Network Open.*

eTable 4. Secondary outcomes

Outcome	Group	Baseline (ITT)	Follow-up (mITT)
**Bowel measures**			
Constipation (≥2 RomeIV), n/N (%)	Mono	48/48 (100)	20/42 (48)
	Comb	46/46 (100)	21/41 (51)
Fecal incontinence, n/N (%)	Mono	32/48 (67)	11/42 (26)
	Comb	40/46 (87)	19/41 (46)
Rectal diameter, median (IQR)	Mono	3.5 (3.2–3.7)	2.9 (2.7–3.7)
	Comb	3.3 (3.0–3.8)	3.0 (2.7–3.5)
Bristol stool scale, median (IQR)	Mono	3.0 (3.0–4.0)	4 (4.0–4.0)
	Comb	3.5 (3.0–4.0)	4 (4.0–4.0)
**Bladder symptoms**			
Urge, n/N (%)	Mono	41/48 (85)	28/41 (68)
	Comb	36/43 (84)	28/39 (68)
**Frequency volume chart**			
Fluid intake, mean ± SD	Mono	1045 ± 310	1023 ± 292
	Comb	988 ± 315	1118 ± 416
Voiding frequency, median (IQR)	Mono	7.0 (5.5–8.0)	6.0 (5.0–7.3)
	Comb	7.5 (5.6–8.5)	7.0 (5.9–8.5)
MVV/EBC, median (IQR)	Mono	72 (59–90)	65 (59–86)
	Comb	57 (48–77)	61 (48–82)

Note. Data are presented as n/N (%) for categorical variables, median (interquartile range, IQR) for non-normally distributed continuous variables, and mean ± SD for normally distributed continuous variables. Baseline and follow-up numbers reflect participants remaining in the study at each time point. Secondary outcomes are presented descriptively; no formal statistical comparisons were performed. MVV = maximal voided volume; EBC = expected bladder capacity; Mono = monotherapy group; Comb = combination therapy group.

© 2026 Axelgaard S et al. *JAMA Network Open.*

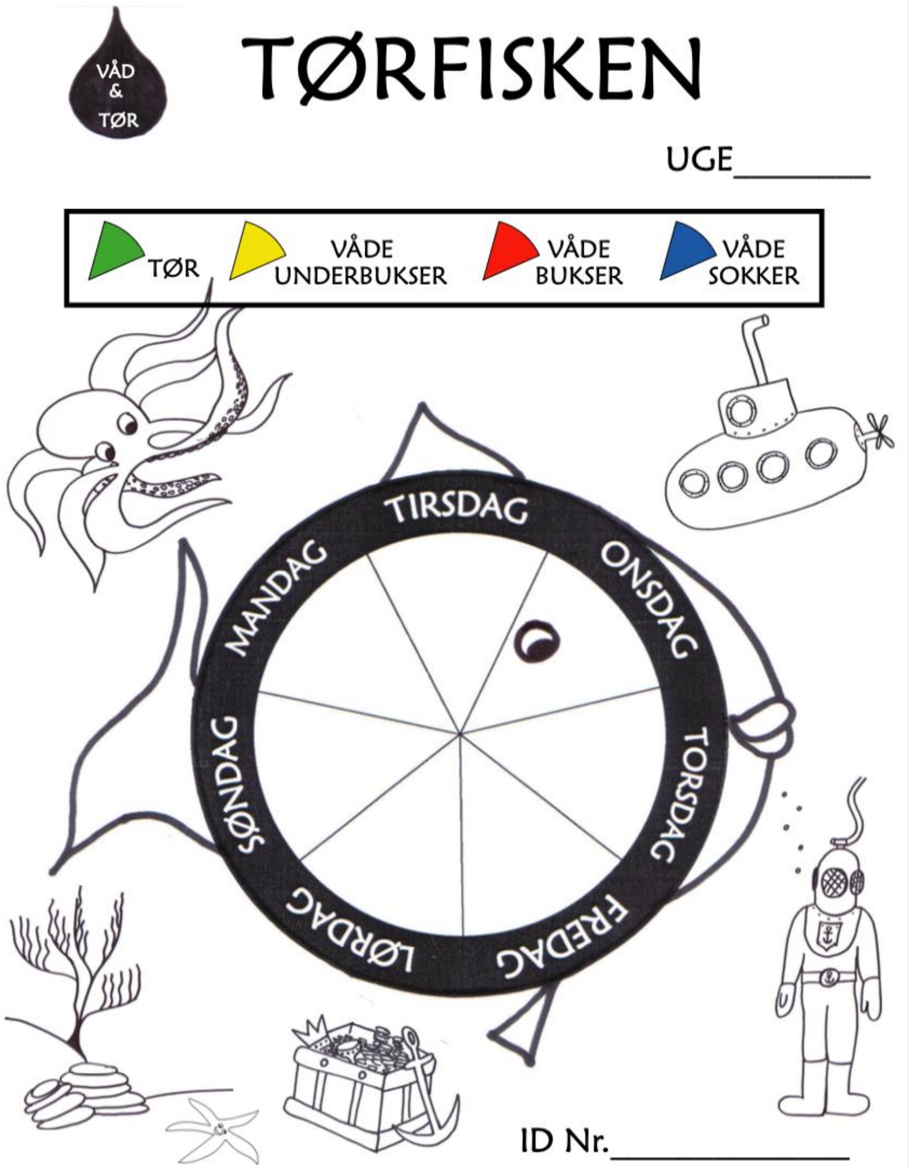

eFigure. Dry Pie bladder diary

Legend. The Danish version of the Dry Pie bladder diary. Participants completed the diary for one week prior to each study visit (baseline and follow-up), with age-appropriate assistance from caregivers. Each day was color-coded to indicate the most severe episode of daytime urinary incontinence: dry, underwear wet, clothes wet, or socks wet. The figure illustrates individual patterns of incontinence over the week.

© 2026 Axelgaard S et al. *JAMA Network Open.*

References for Online-Only Supplement

1. Borch L, Hagstroem S, Bower WF, Siggaard Rittig C, Rittig S. Bladder and bowel dysfunction and the resolution of urinary incontinence with successful management of bowel symptoms in children. Acta Paediatr. 2013;102(5):e215-20.
2. Hagstroem S, Rittig N, Kamperis K, Mikkelsen MM, Rittig S, Djurhuus JC. Treatment outcome of day-time urinary incontinence in children. Scand J Urol Nephrol. 2008;42(6):528-33.
3. Hopewell S, Chan AW, Collins GS, Hróbjartsson A, Moher D, Schulz KF, et al. CONSORT 2025 statement: updated guideline for reporting randomised trials. Bmj. 2025;389:e081123.

© 2026 Axelgaard S et al. *JAMA Network Open.*
