# Supplementary material for: Bowel Management and Standard Urotherapy in Pediatric Bladder and Bowel Dysfunction: A Randomized Clinical Trial
Source: JAMA Netw Open. 2026 Apr 27;9(4):e268836. doi: 10.1001/jamanetworkopen.2026.8836 (PMC13122393; doi:10.1001/jamanetworkopen.2026.8836)
Supplement: Supplement 2. — Trial Protocol and Statistical Analysis Plan [file jamanetwopen-e268836-s002.pdf]

Study Protocol

Title: Bowel Management and Standard Urotherapy in Bladder and Bowel Dysfunction: A Randomized Clinical Trial

Background and Rationale

Bladder and bowel dysfunction (BBD) is defined by the coexistence of functional constipation and lower urinary tract symptoms (LUTS), including daytime urinary incontinence (DUI) (1-3). BBD affects up to 20% of school-aged children (4) and is associated with urinary tract infections (5, 6), renal sequelae (7), and psychosocial burden (8, 9). Current management typically follows a stepwise strategy, beginning with bowel management prior to bladder-directed treatments (10). However, evidence guiding the optimal sequencing of interventions is limited.

This protocol describes a randomized controlled trial evaluating whether adding standard urotherapy (SU) to bowel management improves DUI in children with treatment-naïve BBD.

Objectives

Primary Objective

To determine whether combining SU with bowel management reduces the number of wet days per week compared with bowel management alone.

Secondary Objectives

1. To compare changes in incontinence severity.
2. To assess changes in bowel outcomes (Rome IV, Bristol Stool Scale, transabdominal rectal diameter).
3. To evaluate bladder parameters including voiding frequency and maximum voided volume relative to expected bladder capacity.
4. To explore the effect of bowel management success on DUI outcomes.
5. To assess the association between adherence to SU and treatment response.

Trial Design

Multicentre, open-label, parallel-group randomized controlled trial. Participants will be randomized 1:1 to bowel management alone or bowel management plus SU. Follow-up duration: 12 weeks.

Study Setting

Five paediatric outpatient clinics in Denmark: - Aarhus University Hospital - Aalborg University Hospital - Gødstrup Hospital - Viborg Regional Hospital - Kolding Hospital

Eligibility Criteria

Inclusion Criteria

- Age 5–14 years.
- Treatment-naïve BBD defined as:
    - Functional constipation fulfilling ≥2 Rome IV criteria for ≥1 month.
    - Daytime urinary incontinence ≥2 times weekly for ≥1 month.

Exclusion Criteria

- Anatomical or neurological abnormalities affecting urinary or gastrointestinal function.
- Previous or ongoing urotherapy.
- Previous or current pharmacological treatment for DUI.

Interventions

Bowel Management (Both Groups)

Delivered according to ESPGHAN guidelines (11) and tailored individually. - **Disimpaction:** PEG 3350 (Movicol/Dulcosoft) or sodium picosulfate if PEG not tolerated. - **Maintenance therapy:** PEG 0.5–0.8 g/kg/day or magnesium hydroxide 500–1000 mg/day. Dose adjustments based on response. - **Adjuncts:** Stimulant laxatives or transanal irrigation when indicated. - **Behavioural measures:** Scheduled toileting after meals (1–3 times daily).

Adherence will be reinforced and dosage adjusted via phone consultations at weeks 2 and 4.

Standard Urotherapy (Combination Group Only)

- Education on LUTS mechanisms.
- Timed voiding every 2 hours or adjusted to the child's needs using a Garmin Vivofit jr. 2 timer watch provided at inclusion.
- Guidance on optimal toilet posture.
- Recommended fluid intake of 1200–1500 ml/day.
- Reinforcement during telephone follow-ups.

Outcomes

Primary Outcome

- Number of wet days per week assessed using the Dry Pie bladder diary.
- Analyzed as:
    1. Daily risk of a wet day.
    2. Expected number of wet days per week.
    3. ICCS response categories: no response (<50% reduction), partial response (50–99%), complete response (100%).

Secondary Outcomes

- Incontinence severity score derived from the Dry Pie bladder diary.
- Stool consistency (Bristol Stool Scale).
- Rectal diameter (ultrasound).
- Number of fulfilled Rome IV criteria.
- Fecal incontinence.
- Voiding frequency
- MVV/EBC.

Post-Hoc Outcomes

1. Comparison of DUI outcomes according to bowel management response.
2. Effect of SU adherence.

Sample Size Calculation

Based on expected complete response rates (12, 13), 82 participants (41 per arm) were required to detect a clinically meaningful difference with 80% power and α=0.05. To allow for attrition, we aim at including 100.

Randomisation and Allocation Concealment

Block randomisation (1:1) stratified by site using REDCap. Allocation sequence concealed electronically. Due to intervention type, blinding of participants and providers will not be feasible. Outcome assessors and data analysts will not be blinded.

Data Collection and Follow-Up

- Baseline evaluation: bowel and bladder history, rectal ultrasound, bladder diary.
- Follow-up at 12 weeks: repeat assessments.
- Adverse events will be monitored as part of routine clinical practice.

Statistical Analysis

- Modified intention-to-treat including all participants with available follow-up data.
- Daily risk will be modelled using binomial regression with robust variance clustered by participant.
- Continuous outcomes will be analyzed using ANCOVA adjusting for baseline.
- Categorical outcomes will be compared using χ^2 tests.
- Missing data will not be imputed.
- Significance threshold: $p<0.05$.

Ethics and Registration

Approved by the Regional Committee on Health Research Ethics of the Central Denmark Region (VEK-1-10-72-366-21). Registered at ClinicalTrials.gov (NCT05318365). Written informed consent will be obtained from all parents/legal guardians.

Dissemination

Results will be submitted to peer-reviewed journals and presented at paediatric urology and gastroenterology conferences.

Appendices: - Full Eligibility Criteria (eTable 1) - CONSORT 2025 Checklist (eTable 2) - Statistical Analysis Plan

1. Santos JD, Lopes RI, Koyle MA. Bladder and bowel dysfunction in children: An update on the diagnosis and treatment of a common, but underdiagnosed pediatric problem. Can Urol Assoc J. 2017;11(1-2Suppl1):S64-S72.
2. Koff SA, Wagner TT, Jayanthi VR. The relationship among dysfunctional elimination syndromes, primary vesicoureteral reflux and urinary tract infections in children. J Urol. 1998;160(3 Pt 2):1019-22.
3. Austin PF, Bauer SB, Bower W, Chase J, Franco I, Hoebeke P, et al. The standardization of terminology of lower urinary tract function in children and adolescents: Update report from the standardization committee of the International Children's Continence Society. Neurourol Urodyn. 2016;35(4):471-81.
4. Shaikh N, Hoberman A, Wise B, Kurs-Lasky M, Kearney D, Naylor S, et al. Dysfunctional elimination syndrome: is it related to urinary tract infection or vesicoureteral reflux diagnosed early in life? Pediatrics. 2003;112(5):1134-7.
5. Shaikh N, Hoberman A, Keren R, Gotman N, Docimo SG, Mathews R, et al. Recurrent Urinary Tract Infections in Children With Bladder and Bowel Dysfunction. Pediatrics. 2016;137(1).
6. Sjöström S, Sillén U, Bachelard M, Johansson E, Brandström P, Hellström AL, et al. Bladder/bowel dysfunction in pre-school children following febrile urinary tract infection in infancy. Pediatr Nephrol. 2021;36(6):1489-97.
7. Breinbjerg A, Jørgensen CS, Frøkiær J, Tullus K, Kamperis K, Rittig S. Risk factors for kidney scarring and vesicoureteral reflux in 421 children after their first acute pyelonephritis, and appraisal of international guidelines. Pediatr Nephrol. 2021;36(9):2777-87.
8. Ikeda H, Ono T, Oyake C, Oonuki Y, Watanabe Y, Watanabe T. Comparative analysis of health-related quality of life between children with bladder and bowel dysfunction versus lower urinary tract dysfunction and healthy controls. Investig Clin Urol. 2024;65(5):494-500.
9. Collis D, Kennedy-Behr A, Kearney L. The impact of bowel and bladder problems on children's quality of life and their parents: A scoping review. Child Care Health Dev. 2019;45(1):1-14.

10. Tekgul S, Stein R, Bogaert G, Undre S, Nijman RJM, Quaedackers J, et al. EAU-ESPU guidelines recommendations for daytime lower urinary tract conditions in children. Eur J Pediatr. 2020;179(7):1069-77.
11. Gordon M, de Geus A, Banasiuk M, Benninga MA, Borrelli O, Boruta M, et al. ESPGHAN and NASPGHAN 2024 protocol for paediatric functional constipation treatment guidelines (standard operating procedure). BMJ Paediatr Open. 2025;9(1).
12. Borch L, Hagstroem S, Bower WF, Siggaard Rittig C, Rittig S. Bladder and bowel dysfunction and the resolution of urinary incontinence with successful management of bowel symptoms in children. Acta Paediatr. 2013;102(5):e215-20.
13. Hagstroem S, Rittig N, Kamperis K, Mikkelsen MM, Rittig S, Djurhuus JC. Treatment outcome of day-time urinary incontinence in children. Scand J Urol Nephrol. 2008;42(6):528-33.

STATISTICAL ANALYSIS PLAN (SAP)

Randomized Controlled Trial on Constipation Treatment Alone vs Combination Therapy in Children with Bladder and Bowel Dysfunction (BBD)

---

1. Objectives

Primary objective

- To determine whether bowel management alone reduces daytime urinary incontinence (DUI) compared with bowel management combined with standard urotherapy.

Secondary objectives

1. To compare complete response rates (100% dry days) between groups at follow-up.
2. To evaluate other bladder outcomes (voiding frequency, urgency, maximum voided volume [MVV]/expected bladder capacity [EBC]).
3. To evaluate bowel outcomes (Rome IV, stool consistency using Bristol Stool Scale, transabdominal rectal diameter).
4. To assess changes in incontinence severity scores from baseline to follow-up.
5. To describe adverse events occurring during the intervention period.

---

2. Study Design and Populations

- Parallel group randomized controlled trial with 1:1 allocation.

Analysis populations

- **Modified intention-to-treat (mITT):** All randomized participants with available follow-up data, analyzed according to randomization.
- **Safety population:** All participants who initiated any part of allocated treatment.

---

3. Primary Outcome

Overall outcome: Number of wet days per week

The primary outcome is reduction in DUI, expressed in three complementary ways:

1. **Expected daily risk of DUI:**
    - Modelled as a binary daily measure using binomial regression with log link and robust variance clustered by participant.
    - Effect measure: risk ratio with 95% CI.
2. **Expected number of wet days per week:**
    - Calculated from the daily risk model for each participant.
    - Summarized by treatment group as mean with 95% CI.
3. **Response (≥50% reduction in wet days):**
    - Proportion of participants achieving ≥50% reduction in wet days from baseline to follow-up.
    - Compared between groups using χ^2-test; relative risk with 95% CI.

---

4. Secondary Outcomes

- Complete response rate (100% dry days during final week).
- Change in incontinence severity score (baseline to follow-up).
- Other bladder outcomes: voiding frequency, urgency, MVV/EBC.
- Other bowel outcomes: Rome IV criteria, stool consistency, transabdominal rectal diameter.
- Adverse events monitored during routine clinical care.

Note: Secondary bladder and bowel outcomes will be summarized descriptively only (counts, percentages, mean ± SD, median (IQR)).

---

5. General Analytical Principles

- Two-sided $\alpha = 0.05$.
- 95% confidence intervals reported.
- No adjustment for multiple comparisons.
- Analyses conducted in R.

---

6. Descriptive Statistics

- **Baseline variables:**
    - Continuous: assessed for normality (Q–Q plots); reported as mean ± SD or median (IQR).
    - Categorical: counts and percentages.
- No hypothesis testing for baseline differences.

7. Primary Outcome Analysis

- **Expected daily risk:** Binomial regression with log link, robust variance clustered by participant; risk ratio with 95% CI.
- **Expected number of wet days per week:** calculated from model; summarized by group (mean, 95% CI).
- **Response (≥50% reduction in wet days):** proportion compared using χ^2-test; relative risk with 95% CI.

8. Secondary Outcome Analyses

- **Complete response rate:** χ^2-test; relative risk with 95% CI.
- **Incontinence severity score:** ANCOVA with follow-up score as outcome, baseline score as covariate; adjusted mean difference with 95% CI.
- **Other bladder and bowel outcomes:** summarized descriptively (counts, percentages, medians, means ± SD/IQR).
- **Adverse events:** summarized descriptively.

9. Missing Data

- No imputation; analyses conducted on available cases.

10. Sample Size Calculation

- Based on prior reported response rates, 82 participants (41 per group) required to achieve 80% power with $\alpha = 0.05$ to detect a clinically meaningful difference in complete dryness rates.
- Planned enrolment: 100 participants to allow for dropouts.

11. Deviations from the SAP

- Exploratory post hoc assessments of treatment adherence were conducted; not prespecified.
