# Supplementary material for: Bowel Management and Standard Urotherapy in Pediatric Bladder and Bowel Dysfunction: A Randomized Clinical Trial
Source: JAMA Netw Open. 2026 Apr 27;9(4):e268836. doi: 10.1001/jamanetworkopen.2026.8836 (PMC13122393; doi:10.1001/jamanetworkopen.2026.8836)
Supplement: Supplement 3. — Data Sharing Statement [file jamanetwopen-e268836-s003.pdf]

Data Sharing Statement

Axelgaard. Bowel Management and Standard Urotherapy in Pediatric Bladder and Bowel Dysfunction. *JAMA Netw Open*. Published April 27, 2026.
doi:10.1001/jamanetworkopen.2026.8836

Data
Additional Information: ClinicalTrials.gov NCT05318365
Data available: Yes
Data types: Deidentified participant data, Data dictionary
How to access data: De-identified participant data and the data dictionary will be made available upon reasonable request to the corresponding author at sofiaxel@rm.dk. Requests will be considered after publication and subject to approval of a data use agreement.
When available: With publication

Supporting Documents
Document types: Statistical/analytic code, Informed consent form
How to access documents: Statistical/analytic code and the informed consent form will be made available upon reasonable request to the corresponding author at sofiaxel@rm.dk. Requests will be considered after publication and subject to approval.
When available: With publication

Additional Information
Who can access the data: De-identified participant data, data dictionary, statistical/analytic code, and the informed consent form will be made available to researchers whose proposed use of the data has been approved by the corresponding author.
Types of analyses: The data will be made available for scientific research related to paediatric bladder and bowel dysfunction or related urological/gastroenterological studies, subject to approval by the corresponding author.
Mechanisms of data availability: Data will be made available after approval of a proposal by the corresponding author and subject to a signed data use agreement. The corresponding author will provide support as needed for understanding the dataset and data dictionary.
